# Case Report: Diffuse Large B Cell Lymphoma After Cardiac Transplantation due to Anthracycline-Induced Dilated Cardiomyopathy in Pediatric Acute Lymphoblastic Leukemia

**DOI:** 10.3389/fphar.2022.769751

**Published:** 2022-04-20

**Authors:** Ning Xin, Cao Chunyan, Zhou You, Peng Lu, Jin Runming, Zhou Fen

**Affiliations:** ^1^ Department of Pediatrics, Wuhan Union Hospital, Tongji Medical College, Huazhong University of Science and Technology, Wuhan, China; ^2^ Department of Ultrasound, Wuhan Union Hospital, Tongji Medical College, Huazhong University of Science and Technology, Wuhan, China; ^3^ Department of Pathology, Wuhan Union Hospital, Tongji Medical College, Huazhong University of Science and Technology, Wuhan, China; ^4^ Department of Intensive Care Unit, Sun Yat-sen Memorial Hospital, Sun Yat-sen University, Guangzhou, China

**Keywords:** anthracycline-induced cardiotoxicity, childhood acute lymphoblastic leukemia, dilated cardiomyopathy, cardiac transplantation, diffuse large B cell lymphoma

## Abstract

Anthracycline is a first-line chemotherapy drug used to treat childhood acute leukemia, which may cause cardiac toxicity including common arrhythmia, valve disease, pericardial effusion, and even rare cardiomyopathy and cardiac failure. We reported a 2-year-old boy who was treated irregularly for acute lymphoblastic leukemia with daunorubicin. After 26 months, his left ventricular ejection fraction decreased to 40% and progressively decreased to 20–30%. Then he successfully received a heart transplant and the myocardium was confirmed with dilated cardiomyopathy. Eight months after cardiac transplantation, he was admitted again for left neck mass and was diagnosed with monomorphic diffuse large B cell lymphoma associated with Epstein-Barr virus infection by biopsy. We present this case to highlight the importance of standard chemotherapy of daunorubicin, clinical prevention, and monitoring of anthracycline-induced cardiotoxicity in acute lymphoblastic leukemia children to ensure their good prognosis and long-term life quality.

## Introduction

It is well known that cardiotoxicity is a main side effect of anthracyclines. The cardiotoxicity tends to great inter-individual variability in severity, ranging from asymptomatic structural changes on cardiac imaging, to new onset arrhythmia, to dilated cardiomyopathy or refractory heart failure (Giantris et al., 1998; Kremer et al., 2002; Grande et al., 2003). Severe cardiotoxicity (grade 4) such as heart failure or dilated cardiomyopathy caused by chemotherapeutic agents is rare, as chemotherapy for children with acute lymphoblastic leukemia (ALL) has become increasingly standardized and dosage of daunorubicin has been limited. There is currently no effective treatment for severe cardiotoxicity except heart transplantation, which also carries a risk of graft vs. host disease (GVHD) or post-transplant lymphocytic proliferation diseases.

We reported a rare case of B-ALL child developing heart failure, dilated cardiomyopathy after administering the cumulative dosage of 410 mg/m^2^ DNR and secondary diffuse large B cell lymphoma after heart transplantation.

### Case Presentation

**FIGURE 1:**
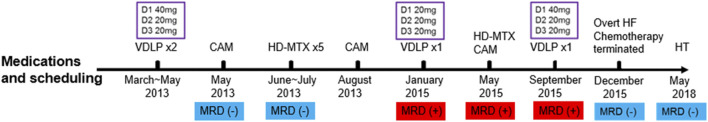
The detailed information of chemotherapy and MRD; VDLP: vindesine (VDS), daunorubicin (DNR), l-asparaginase, and prednisone; CAM: cyclophosphamide (CTX), cytarabine (Ara-C), and mercaptopurine (6-MP). HD-MTX: high dose of methotrexate; HF: heart failure; HT: heart transplantation. The purple boxes revealed daunorubicin infusion dosages from Day 1 (D1) to Day 3 (D3).

A 2-year-old boy was admitted in February 2013 for looking pale more than 20 days and was diagnosed as B-type acute lymphoblastic leukemia (ALL) (TEL/AML1 positive, low risk) in a local hospital where he received a non-standard chemotherapy protocol with alarmingly high doses of daunorubicin (DNR). The detailed information of chemical drugs and minimal residual disease (MRD) are showed in Figure 1. He was administrated two courses of VDLP (including vindesine, DNR, l-asparaginase, and prednisone) as induction therapy. Another two courses of VDLP were given during the maintenance phase because of positive MRD (0.55%). DNR was administrated three consecutive daily doses every 3 weeks and the infusion dosage each day is presented in Figure 1. The cumulative dosage of DNR was 410 mg/m^2^.

**FIGURE 2:**
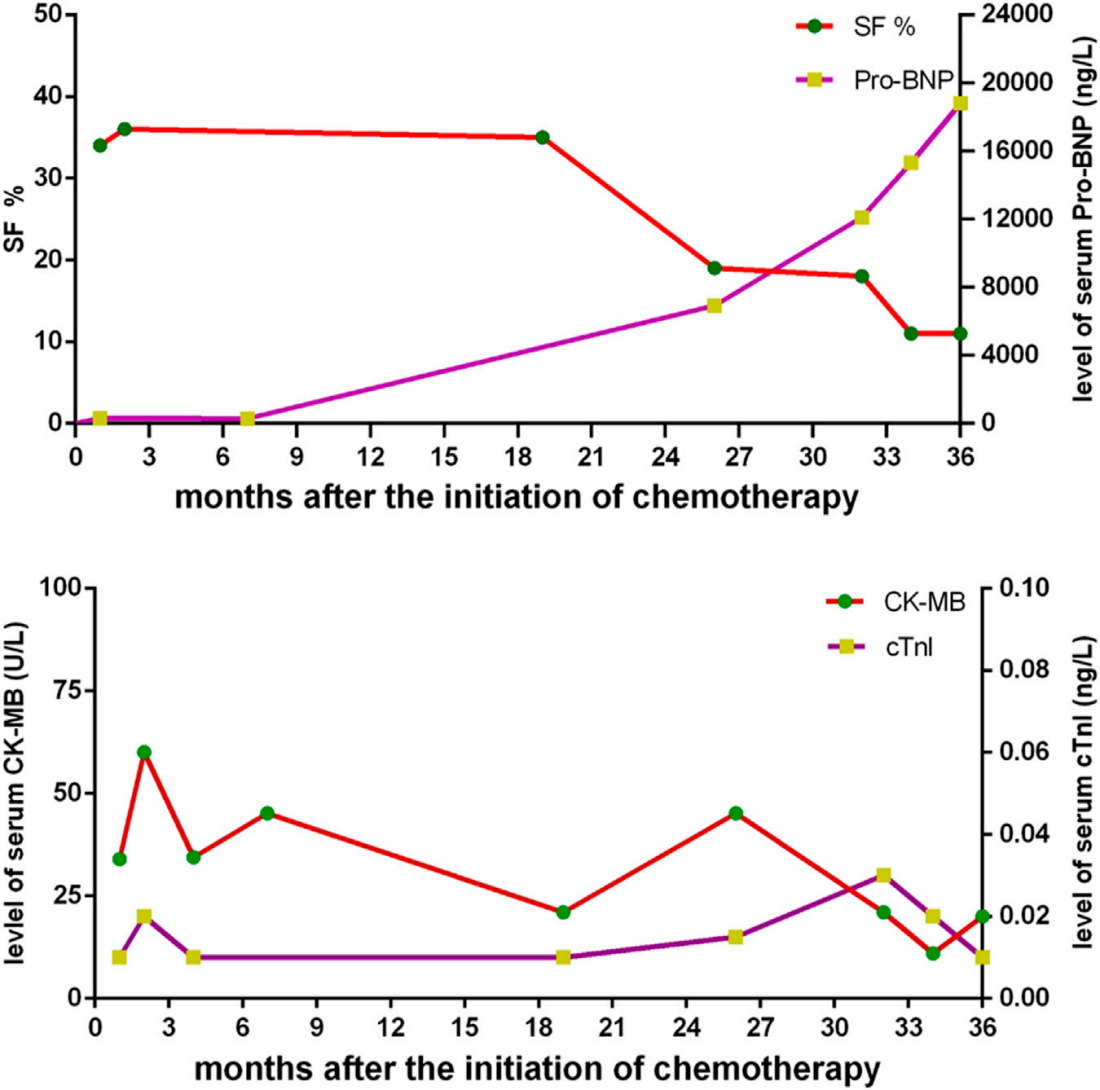
The changes of shortening fraction (SF), serum pro B type natriuretic peptide (Pro-BNP), creatine kinase (CK-MB), and cardiac troponin I (cTnI) after the outset of chemotherapy.

**FIGURE 3:**
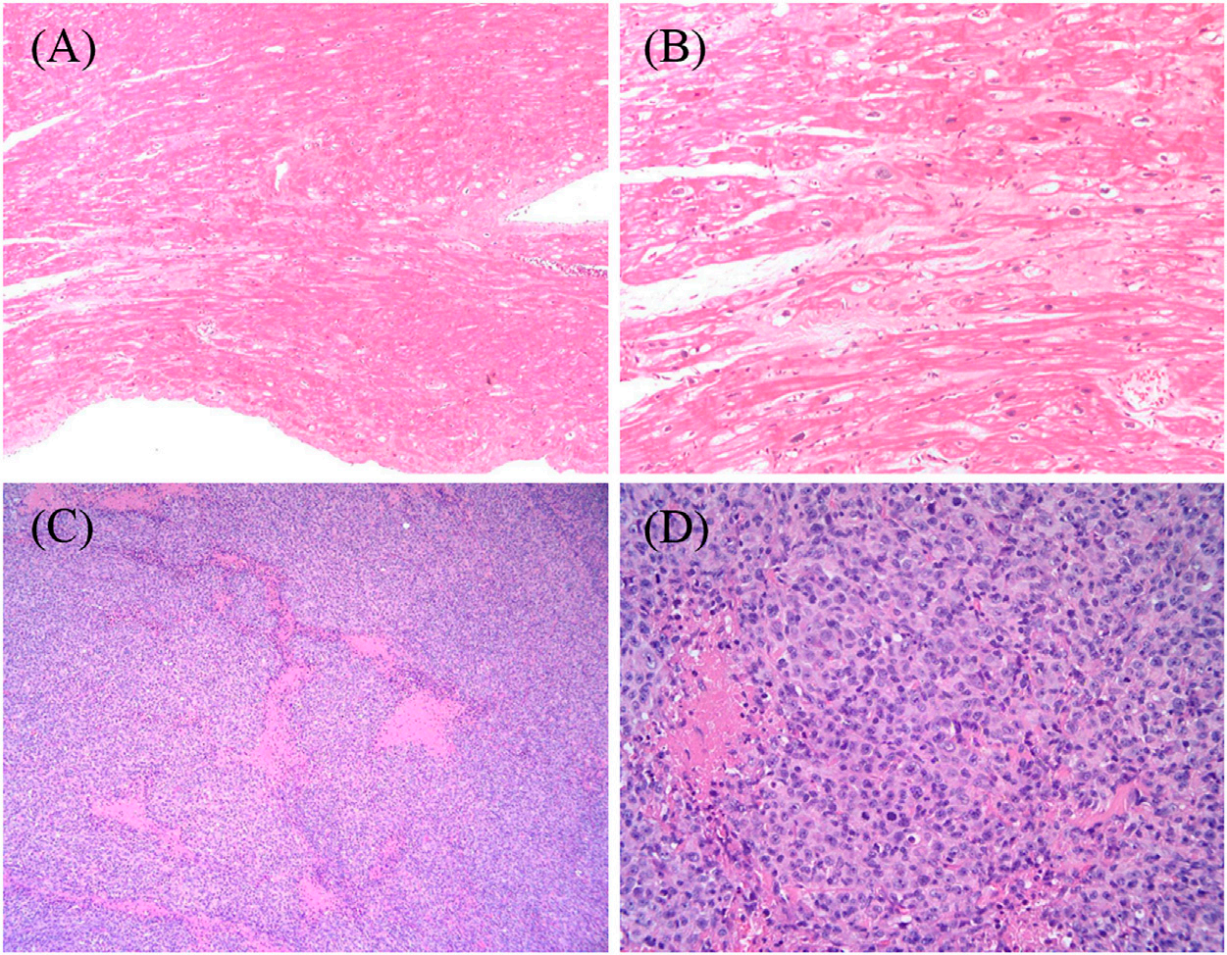
**(A,B)** The structural changes of this transplant recipient’s myocardium, being magnified respectively×100 and ×400 by light microscope. The myocardium of the specimen from the heart transplant recipient revealed hypertrophy of cardiomyocytes with disordered arrangement; segmental vacuolar degeneration; and myocardial fibers blurring. **(C,D)** The histopathological characteristics of the biopsy tissue from retroauricular lymph nodes on the left side, being magnified respectively×100 and ×400 by light microscope. The images show groups of lymphoma cells compartmentalized by fine fibrosis, large and arranged neoplasm in a diffuse pattern that partially effaces normal nodal architecture, and areas of geographic necrosis. The lymphoma cells are moderately large in size with an abundant amount of cytoplasm, most of which is without nucleoli.

A routine follow-up examination of echocardiography found a decreased left ventricular ejection fraction (LVEF) approximately 40% at 26 months after chemotherapy, and the child had no clinical manifestations of cardiac dysfunction. With administrating another dose of daunorubicin, his cardiac failure was aggravated. He was admitted again for abdominal pain and then heart failure (HF) symptoms such as cough, arduous asthma, orthopnea, and hepatomegaly gradually presented. Though chemotherapy was terminated, and medications of digoxin, coenzyme, captopril, and diuretics were prescribed, LVEF was progressively decreased even to 20–25%. Shortening fraction (SF), serum pro B type natriuretic peptide (Pro-BNP), creatine kinase (CK-MB), and cardiac troponin I (cTnI) were also routinely monitored and are shown in Figure 2. In May 2018, he received cardiac transplantation in our hospital. The pathology of myocardial tissue suggested dilated cardiomyopathy seen in Figures 3A,B. Then his cardiac function and laboratory parameters gradually recovered to normal range, and he was discharged in a good condition.

After transplantation, immunosuppressive agents such as tacrolimus and mycophenolate mofetil, and ganciclovir prophylaxis for cytomegalovirus were administrated. The blood levels of tacrolimus and mycophenolate mofetil were monitored regularly in target levels. Seven months after transplantation, this patient was hospitalized for a left neck mass in January 2019. The biopsy confirmed monomorphic diffused large B cell lymphoma (DLBCL) associated with Epstein-Barr virus (EBV) as shown in Figures 3C,D. The viral load for EBV in whole blood and plasma measured by quantitative real-time polymerase chain reaction was 4190 copies/ml. He received standard R-CHOP therapy combined with radiotherapy and ganciclovir since March 26, 2020 and now is in complete remission.

To understand the genetic status of this patient, we performed whole exome sequencing and focused on the tumor susceptibility gene and hereditary dilated cardiomyopathy gene. The result is negative. The detection of drug gene polymorphism showed genetic variation of SLC28A3 C1381T CC type, which means a higher risk of anthracycline-induced cardiotoxicity (ACT).

## Discussion

In general, there is a long latency period between anthracycline exposure and symptomatic cardiotoxicity, and cardiotoxicity is difficult to clinically observe in its early stage, especially in children. Once it is symptomatic, there is often limited time left to try different treatment strategies, resulting in short-lived outcomes. In our report, this patient appeared asymptomatic LVEF decrease in the interval between the third dose and fourth dose of DNR during chemotherapy and progressed rapidly toward symptomatic heart failure requiring transplantation.

The secondary non-Hodgkin’s lymphoma (NHL) is an exceedingly rare complication following chemotherapy for ALL, and its incidence was about 0.08% within 10 years (Löning et al., 2000). DLBCL was reported to be the subtype that occurred most often after treatment for childhood cancer. One of the prominent factors is immunosuppression. In this case, the patient received heart transplantation after ACT. Exposure to induction therapy and intensive immunosuppressive regimen after heart transplantation concomitant with EBV infection are the origin that exacerbates the development of secondary primary DLBCL. Although ACT and post-transplant DLBCL have been widely studied and reported, it is relatively rare that the overdose of DNR, ACT, and post-transplant secondary lymphoma unexpectedly occur in this patient, which may be avoidable. Therefore, the safe dose of DNR, subclinical identification, and management of potential toxicity based on standard chemotherapy for children with ALL should be highlighted to avoid poor outcomes.

Early-onset DLBCL is the most life-threatening complication, which usually occurs in the first year after heart transplantation due to intensive immunosuppression and EBV infection. Except for immunosuppression and EBV infection, IgH and IgK rearrangement may be a factor contributing to the etiology of early-onset DLBCL in this patient. At diagnosis of this child, IgH and IgK rearrangement was positively detected. It was confirmed that IgH and IgK were mainly found in marginal zone B-cell lymphomas of mucosa lymphoid tissue (MALT lymphomas), DLBCL, and mantle cell lymphomas (Bo et al., 2016; Zhang et al., 2016; Lu et al., 2017). The sequencing of immunoglobulin rearrangement in either cellular or circulating extracellular DNA of DLBCL can be used as a sensitive predictor of achieving remission and relapse in the molecular level (Kurtz et al., 2015). It is reasonable to infer that this lymphocyte cloning hyperplasia with gene rearrangements might further contribute to elevated risk of neoplastic conversion to DLBCL among heart transplant recipients, in particular when immunosuppressive regimen facilitates EBV infection and hinders cytotoxic T-cell immunity.

Heart transplantation and related complications may influence the outcome of ALL children. Therefore, it is especially important to dynamically monitor the earliest cardiac changes in cardiac structure and function and explore timely intervention in children with ALL. First, we should know which patients are at high risk of ACT. Recently, one of the most prominent and well-established risk factors is total accumulative dosage of anthracycline. The risk of cardiomyopathy is especially high in children treated with a cumulative dose of 250 mg/m^2^ or more. Von Hoff (Von Hoff et al., 1979) reported that the cumulative possibility of developing heart failure was 3, 7, and 18% at 400 mg/m^2^, 500 mg/m^2^, and 800 mg/m^2^. Importantly, there appears to be no absolutely safe dose of anthracycline when symptomatic cardiomyopathy has been reported in survivors who received doses well below 250 mg/m^2^ (Oeffinger et al., 2006; Mulrooney et al., 2009; Blanco et al., 2012; van der Pal et al., 2012). Besides, other risk factors such as age under 4 years, schedule of anthracycline administration (the single dose every 3 weeks), longer length of follow-up, female, other cardiotoxic drugs (such as cyclophosphamide), and genetic variations of RARG rs2229774, SLC28A3 rs7853758, and UGT1A6*4 rs17863783 are considered as cofounding variables to identify the increased risk of cardiac injury (Von Hoff et al., 1979; Giantris et al., 1998; Aminkeng et al., 2016). The cumulative dosage of anthracycline this patient received is unusually higher than what children’s ALL treatment protocols recommend as 25 or 50 mg/m^2^ in each VDLP block, which is a mainly attributable factor to his cardiotoxicity. By comparing with other ALL protocols then approved for clinical trials in other parts of China, it seems obvious that this patient received the highest record of DNR cumulative dosage that is highly risky to cardiomyocytes. The patient also received the administration of three consecutive daily doses that adult patients with ALL are subject to. The comparisons of ALL protocols and DNR cumulative dosages are detailed in Supplementary Tables S1 and S2 (Supporting Information 3). Heart damage following the hazardous level of DNR is the culprit of his poor outcome. We present this case to help clinicians and oncologists to keep vigilant for ACT when they potentially prescribe cardiotoxic drugs. The other risks are 2 years old when first diagnosed and form of anthracycline administration (3 consecutive daily doses every 3 weeks but not a weekly dose) and genetic variant of SLC28A3.

The early detection of ACT for patients especially with high risks should be explored. Detailed two-dimensional echocardiography is a strong recommendation to identify cardiac dysfunction in this population. Ejection fraction (EF), SF, and wall stress, which were regularly monitored in the case, are the most frequently used and readily reproducible parameters of left ventricular (LV) systolic function. However, subclinical cardiotoxicity can be detected by several echocardiographic parameters including not only EF, SF, and LV wall stress, but also decreased LV mass, velocity of shortening corrected for heart rate, LV thickness to dimension ratio, and diastolic dysfunction (Kremer et al., 2002; Lipshultz et al., 2005; Hudson et al., 2007). Therefore, echocardiographic surveillance in these patients should be regular and comprehensive. For serum biomarkers, cTnI was reported to be not associated with LV dysfunction. There is evidence to suggest that persistent elevation of serum natriuretic peptides during treatment with anthracycline may be a predictor of cardiac dysfunction years after completion of therapy (Kismet et al., 2004; Soker and Kervancioglu, 2005; Mavinkurve-Groothuis et al., 2009; Lipshultz et al., 2012; Sherief et al., 2012). The profiles of these serum biomarkers in this report are consistent with previous studies.

In conclusion, a combination of risk factors markedly enhances the overall risk of ACT and DLBCL post-transplantation in this patient. The ability to individualize a safe dose of anthracycline, to identify subclinical ACT, and to remain alert about risk factors of the developing of post-transplant lymphoproliferative disorder among transplant recipients remain a great challenge.

## Data Availability

The original contributions presented in the study are included in the article/Supplementary Material, further inquiries can be directed to the corresponding author.
