# Supplementary material for: Case Report: Diffuse Large B Cell Lymphoma After Cardiac Transplantation due to Anthracycline-Induced Dilated Cardiomyopathy in Pediatric Acute Lymphoblastic Leukemia
Source: Front Pharmacol. 2022 Apr 20;13:769751. doi: 10.3389/fphar.2022.769751 (PMC9065553; doi:10.3389/fphar.2022.769751)
Supplement: Supplementary file 1 [file Table1.DOCX]

Supplementary Material

Supporting information 1 CCLG-2015 chemotherapy protocol

| *Treatment Block* | *Low-Risk* |
| --- | --- |
| Induction | **VDLP (5 weeks)**  - Dexamethasone 6mg/m^2^ D1-4  - Prednisolone 45mg/m^2^ D5-28  - L-Asparaginase 6000unit/m^2^ Day 6,8, 10, 12, 14, 16,18,20, 22, 24  - Vincristine 1.5mg/m^2^ D5, 12, 19, 26  - Daunorubicin 25mg/m^2^ D5, 12(2x)  - IT MTX+Ara-C+DXM Triple D5, 19 |
| Early intensification | **CAM (week 5-7)**  - Cyclophophamide 1000mg/m^2^ D29  - Ara-C 50mg/m^2^ D29-35  - 6MP 60mg/m^2^ D29-35  - IT Triple D29 |
| Consolidation | **6-MP+HD-MTX (8 weeks)**  - 6MP 25mg/m^2^ D1-56 HD-MTX 3gm/m^2^ D1, 15, 29, 43  - IT Triple Q2wk with HD-MTX (4x) |
| Interim Maintenance | **6-MP+ MTX /VD+IT (3 weeks cycle, total 2 cycles)**  -6MP 50mg/m^2^/day, D1-21  -MTX 25mg/m^2^/week, D8-21  -Dexamethasone 8mg/m^2^, D1-7, D22-28  -Vincristine 1.5mg/m2 (D1, 22)  -IT triple (D1,22) |
| Re-induction | **Re-induction 1,2 (6 weeks)**  *1 VDLD (3 weeks)*  - Dexamethasone 8mg/m^2^ D1-7, D15-21 (no taper)  - Doxorubicin 25mg/m^2^ D1  - Vincristine 1.5mg/m^2^ D1, 8, 15  - L-Asparaginase 6000units/m^2^ D3, 5, 7, 9,11,13,15,17,19,21  -IT triple, D1  **6-MP+ MTX /VD+IT (4 weeks cycle, total 2 cycles)**  *2 VLD (3 weeks)*  - Dexamethasone 8mg/m^2^ D1-7, D15-21 (no taper)  - Vincristine 1.5mg/m^2^ D1, 8, 15  - L-Asparaginase 6000units/m^2^ D3, 5, 7, 9,11,13,15,17,19,21  -IT triple, D1 |
| Maintenance | **6-MP+MTX /VD+IT (4 weeks cycle, total 5 cycles)**  -6-MP 50mg/m^2^/day D1-28  -MTX 25mg/m^2^/week D1-21  -Dexamethasone 8mg/m^2^ every 4 weeks (D22-28)  -Vincristine 1.5mg/m^2^ every 4 weeks (D22)  -IT Triple every 4 weeks (D22)  **Group A**  **6-MP+MTX /VD+IT (8 weeks cycle, total 7 cycles)**  -6MP 50mg/m^2^/day D1-56  -MTX 25mg/m^2^/week D1-56  -Dexamethasone 8mg/m^2^ every 8 weeks (D50-56)  -Vincristine 1.5mg/m^2^ every 8 weeks (D50)  6-MP+MTX to total therapy duration 2.5 years  **Group B**  **6-MP+MTX to total therapy duration 2.5 years** |

Supporting information 2 CALLG-2008 chemotherapy protocol for adults

| *Treatment Block* | *Low-Risk* |
| --- | --- |
| Induction | **VDCLP**  - Prednisolone 1mg/kg/d, D1-14, taper  - L-Asparaginase 6000unit/m^2^, Day 11, 14, 17,20, 23, 26  - Vincristine 2.0mg/m^2^, D1, 8, 15, 22  - Daunorubicin 40mg/m^2^, D1-3, 15-16  - Cyclophophamide 750mg/m^2^, D1,15  - IT MTX+Ara-C+DXM Triple D5, 19 |
| Early intensification | **CAM**  - Cyclophophamide 750mg/m^2^, D1,8  - Ara-C 100mg/m^2^, D1-3,8-10  - 6MP 60mg/m^2^, D1-7  - IT Triple D29 |
| Consolidation | **HD-MTX+L**  - HD-MTX 3g/m^2^, D1  - L-Asparaginase 6000unit/m^2^, Day 3,4  - IT MTX+DXM, D1  **MA**  - mitoxantrone 8mg/m^2^, D1-3  -Ara-C 0.75g/m^2^,q12h, D1-3 |
| Delayed intensification | **VDLD**  - Dexamethasone 8mg/m^2^, D1-7, D15-21 (no taper)  - Daunorubicin 40mg/m^2^, D1-3  - Vincristine 2.0mg/m^2^, D1, 8, 15,22  - L-Asparaginase 6000units/m^2^, D11,14 ,17,20,23,26  ***COATD***  - Cyclophophamide 750mg/m^2^, D1  - Dexamethasone 6mg/m^2^, D1-7, (no taper)  - Vincristine 2.0mg/m^2^, D1  - Ara-C 100mg/m^2^, D1-7  -Vm26 100mg/m^2^, D1-4  **HD-MTX+L**  **-** HD-MTX 3g/m^2^, D1  - L-Asparaginase 10000unit, Day 3,4  - IT MTX+DXM,D1  **TA**  -Vm26 100mg/m^2^, D1-4  - Ara-C 100mg/m^2^, D1-7 |
| Maintenance | **6-MP+MTX (monthly)**  -6MP 60mg/m^2^/day D1-7  -MTX 20mg/m^2^, D8  **MOACD (every 6 months)**  -mitoxantrone 8mg/m^2^, D1,2  - Vincristine 2.0mg/m^2^, D1  -CTX 600mg/m^2^, D1  -Ara-C 100mg/m^2^, D1-5  -Dexamethasone 6mg/m^2^, D1-7 |

| Chemotherapy protocols(LR) | SCMC-ALL-2005 | CCLG-ALL-2015 | BCH-2003 | CCLG-ALL-2008 | CALLG-2008 | Treatment of this patient |
| --- | --- | --- | --- | --- | --- | --- |
| Induction phase | VDLP | VDLD | VDLP | VDLD | VDCLP | VDLP x2 |
| Consolidation phase |  |  |  |  |  |  |
| Early intensification | / | / | CAM x2 | CAM | CAM | CAM |
| Consolidation | CAM | CAM | 6-MP+HD-MTX(4x) | 6-MP+HD-MTX(4x) | HD-MTX+L+MA | 6MP+HD-MTX(5x) |
| HD-MTX | 6-MP+HD-MTX(5x) | 6-MP+HD-MTX(4x) | / | / | HD-MTX+L | / |
| Re-induction phase |  |  |  |  |  |  |
| Delayed intensification | / | / | VDLD+CAM | VDLD+CAT | VDLD | CAM |
| Re-induction | VDLD+CAM | VDLD | / | / | / | / |
| Interim Maintenance | / | 6-MP+MTX/VD | 6-MP+MTX/VD | / | / | / |
| 2nd Delay Intensification | VAD | / | HD-MTX(2x) | / | COATD+TA | / |
| maintenance | 6-MP+MTX/  6-MP+MTX+VD | 6-MP+MTX/VD+IT | 6-MP+MTX/VD | 6-MP+MTX /VD+IT | 6-MP+MTX  /MOACD | Positive MRD  (VDLP+HD-MTX+CAM+VDLP) |

Supporting information 3-Table 1 the comparisons of ALL protocols for low risk patients（children and adults）in China

LR:low risk; SCML-ALL-2005: Shanghai Children’s Medical center(SCMC) network-based ALL 2005 study^1^;CCLG-ALL-2008/2015:Chinese Childhood leukemia Group(CCLG)-ALL 2008 protocol^2^/2015 protocol(supporting information 1);BCH-2003:Beijing Children’s Hospital(BCH)-2003 protocol^3^;CALLG-2008:Chinese Acute Lymphoblastic Leukemia Group(CALLG)-2008 guideline for adults(supporting information 2); VDLP:vindesine (VDS), DNR, L-asparaginase(L) and prednisone; VDLD: VDS, DNR, L-asparaginase and dexamethasone(DXM); VDCLP:VDS, DNR, cyclophosphamide(CTX),L-asparaginase and prednisone; CAM: CTX, cytarabine (Ara-C) and mercaptopurine (6-MP); HD-MTX:high dose of methotrexate; MA: mitoxantrone and Ara-C; CAT:CTX, Ara-C and 6-TG; VD:VDS and DXM; VAD:VDS, Ara-C and DXM; COATD:CTX,VCR,Ara-C,Vm26 and DXM; TA:Vm26 and Ara-C; IT: intrathecal injection; MOCAD:mitoxantrone,VDS,CTX,Ara-C and DXM.

| Chemotherapy protocol(LR) | CCLG-ALL-2015 | SCMC-ALL-2005 | CCLG-ALL-2008 | BCH-2003 | CALLG-2008 | Treatment of this patient |
| --- | --- | --- | --- | --- | --- | --- |
| Induction | Daunorubicin  25mg/m^2^ D5, 12 | Daunorubicin  25mg/m^2^ D8, 15 | Daunorubicin  25mg/m^2^ D8, 15 | Daunorubicin  30mg/m^2^ D8, 15 | Daunorubicin  40mg/m^2^ D1-3, D15-16 | Daunorubicin  (60mg/m^2^ D8,30mg/m^2^ D9-10) x2 |
| Re-induction | Daunorubicin  25mg/m^2^ D1 | Daunorubicin  25mg/m^2^ D1 | / | / | / | / |
| Delayed intensification | / | / | Doxorubicin  25mg/m^2^ D1, 8, 15 | Doxorubicin  30mg/m^2^ D8, 15, 22, 29 | Daunorubicin  40mg/m^2^ D1-3 | / |
| Maintenance | / | / | / | / | / | Daunorubicin  25mg/m^2^ D1-3  45mg/m^2^ D1,25mg/m^2^ D2-3 |
| Cumulative dosages | 75mg/m^2^ | 75mg/m^2^ | 125mg/m^2^ | 180mg/m^2^ | 320mg/m^2^ | 410mg/m^2^ |

Supporting information 3-Table 2 The cumulative dosages of DNR in different chemotherapy protocols

1. Liang Y, Yang LH, Jiang H, et al. Treatment outcome of young children with acute lymphoblastic leukaemia: achievements and directions implied from Shanghai Children's Medical Centre based SCMC-ALL-2005 protocol. *British journal of haematology.* 2015;169(2):267-277.

2. Cui L, Li ZG, Chai YH, et al. Outcome of children with newly diagnosed acute lymphoblastic leukemia treated with CCLG-ALL 2008: The first nation-wide prospective multicenter study in China. *American journal of hematology.* 2018;93(7):913-920.

3. Gao C, Liu SG, Yue ZX, et al. Clinical-biological characteristics and treatment outcomes of pediatric pro-B ALL patients enrolled in BCH-2003 and CCLG-2008 protocol: a study of 121 Chinese children. *Cancer cell international.* 2019;19:293.
