# Supplementary figures and images for: Case Report: Diffuse Large B Cell Lymphoma After Cardiac Transplantation due to Anthracycline-Induced Dilated Cardiomyopathy in Pediatric Acute Lymphoblastic Leukemia
Source: Front Pharmacol. 2022 Apr 20;13:769751. doi: 10.3389/fphar.2022.769751 (PMC9065553; doi:10.3389/fphar.2022.769751)

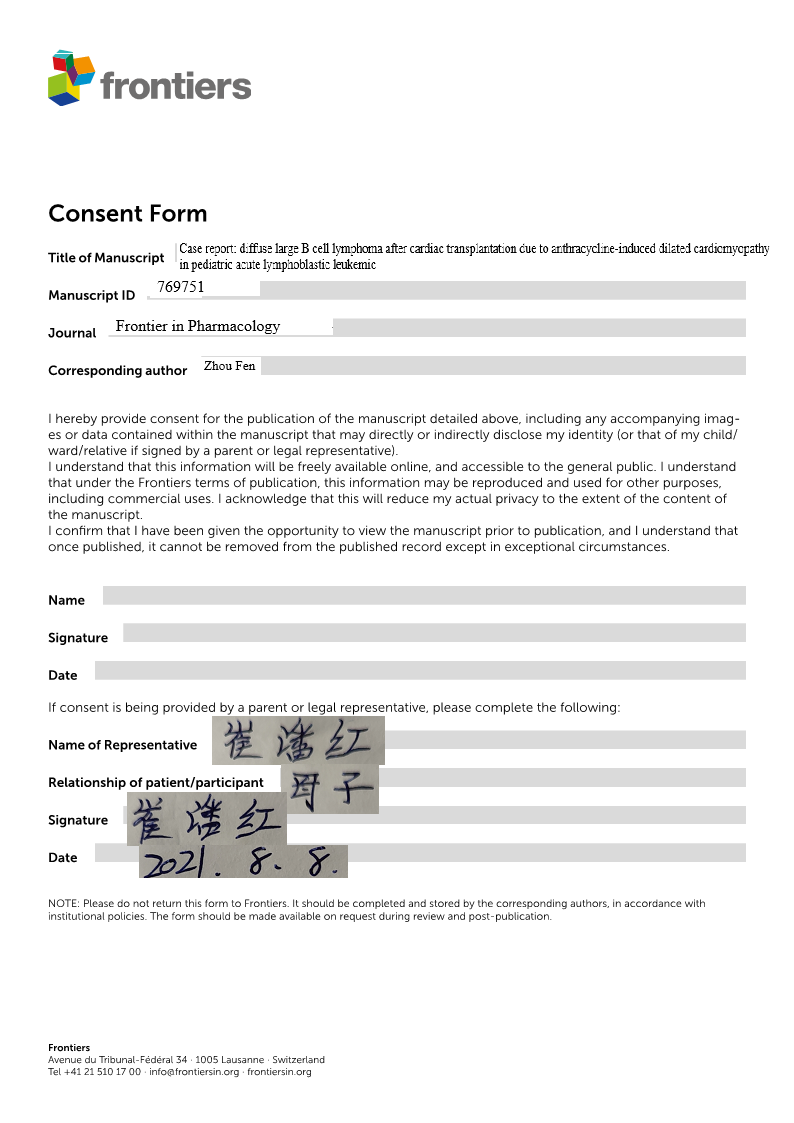

Supplement: Supplementary file 2 [file Image1.PNG]
